# Clinical and molecular characterization of *POU3F4* mutations in multiple DFNX2 Chinese families

**DOI:** 10.1186/s12881-018-0630-9

**Published:** 2018-09-04

**Authors:** Yu Su, Xue Gao, Sha-Sha Huang, Jing-Ning Mao, Bang-Qing Huang, Jian-Dong Zhao, Dong-Yang Kang, Xin Zhang, Pu Dai

**Affiliations:** 1 Department of Otorhinolaryngology, Head and Neck Surgery, PLA General Hospital, Beijing, 100853 People’s Republic of China; 2 Department of Otorhinolaryngology, Hainan Branch of PLA General Hospital, Sanya, 572000 People’s Republic of China; 3 Department of Medical Imaging, PLA 307 Hospital, Beijing, 100074 People’s Republic of China; 4 Department of Otolaryngology, The General Hospital of the PLA Rocket Force, 16# Xi Wai Da Jie, Beijing, 100088 People’s Republic of China

**Keywords:** *POU3F4*, DFNX2, X-linked deafness, Mutation

## Abstract

**Background:**

Many X-linked non-syndromic hearing loss (HL) cases are caused by various mutations in the POU domain class 3 transcription factor 4 (*POU3F4*) gene. This study aimed to identify allelic variants of this gene in two Chinese families displaying X-linked inheritance deafness-2 (DFNX2) and one sporadic case with indefinite inheritance pattern.

**Methods:**

Direct DNA sequencing of the *POU3F4* gene was performed in these families and in 100 Chinese individuals with normal hearing.

**Results:**

There are characteristic imaging findings in DFNX2 Chinese families with *POU3F4* mutations. The temporal bone computed tomography (CT) images of patients with DFNX2 are characterized by a thickened stapes footplate, hypoplasia of the cochlear base, absence of the bony modiolus, and dilated internal acoustic meatus (IAM) as well as by abnormally wide communication between the IAM and the basal turn of the cochlea. We identified three causative mutations in *POU3F4* for three probands and their extended families. In family 1468, we observed a novel deletion mutation, c.973delT, which is predicted to result in a p.Trp325Gly amino acid frameshift. In family 2741, the mutation c.927delCTC was identified, which is predicted to result in the deletion of serine at position 310. In both families, the mutations were located in the POU homeodomain and are predicted to truncate the C-terminus of the POU domain. In the third family, a novel de novo transversion mutation (c.669 T > A) was identified in a 5-year-old boy that resulted in a nonsense mutation (p.Tyr223*). The mutation created a new stop codon and is predicted to result in a truncated POU3F4 protein.

**Conclusions:**

Based on characteristic radiological findings and clinical features, *POU3F4* gene mutation analysis will increase the success rate of stapes operations and cochlear implantations, and improve molecular diagnosis, genetic counseling, and knowledge of the molecular epidemiology of HL among patients with DFNX2.

## Background

Hearing loss (HL) affects 1–3 per 1000 newborns, and the majority of congenital cases of HL are attributable to genetic factors [1]. Previous studies have indicated that deafness is transmitted with an inheritance pattern consistent with autosomal recessive transmission in 75–77% of cases and with autosomal dominant transmission in 15–20% of cases; 2–3% of human hereditary HL is caused by X-linked mutations [2, 3].To date, six deafness loci (DFNX1–6) have been mapped to chromosome X, with five of the corresponding genes identified: *PRPS1* for DFNX1 [4], *POU3F4* for DFNX2 [5], *SMPX* for DFNX4, AIFM1 for DFNX5 [6] and *COL4A6* for DFNX6 [7–9] . *PRPS1* on Xq22 encodes phosphoribosyl pyrophosphate synthetase 1; *POU3F4* on Xq21 encodes a member of a transcription factor family that contains a POU domain; *SMPX* on Xp22 encodes the small muscle protein; and the recently identified gene *COL4A6* encodes the alpha-6 chain of collagen type IV at Xp21.

DFNX2, X-linked deafness type 2, is the most common type of X-linked HL in humans. Mutations in the *POU3F4* gene were first found in 1995 [5] following its chromosomal localization to the X chromosome in 1988 [10]. In fact, deafness caused by *POU3F4* mutation accounts for nearly 50% of all cases of X-linked non-syndromic HL [5, 11]. Clinical features of DFNX2 often include mixed, progressive HL, temporal bone anomalies, and stapes fixation [12, 13]. Affected males exhibit either mixed deafness or, less commonly, only sensorineural deafness. The temporal bone computed tomography (CT) image is characterized by a thickened stapes footplate, hypoplasia of the cochlear base, absence of the bony modiolus, and dilated internal acoustic meatus (IAM), as well as abnormally wide communication between the IAM and the basal turn of the cochlea [14]. Nance et al. first reported X-linked mixed deafness with congenital stapes fixation and a perilymphatic gusher [15]. Sennaroglu et al. suggested that the radiologic phenotype “X-linked deafness with stapes gusher” be called “incomplete partition type III” (IP- III) [16].

To date, nearly 60 different mutations in the coding region of the *POU3F4* gene, including deletions, inversions, and duplications, have been reported to be associated with non-syndromic HL in families with DFNX2 (Table 1). Several reports have described mutations of *POU3F4* in patients with HL and temporal bone abnormalities. In this report, we describe the clinical features and genetic analysis of two Chinese families displaying X-linked inheritance HL and one sporadic case with indefinite inheritance pattern. Moreover, two novel mutations (including a de novo mutation) were identified in the POU-specific and homeodomains of *POU3F4*, coinciding with familial HL.

**Table 1:** Reported Mutations in the *POU3F4* Gene Resulting in DFNX2 Phenotypes

Nucleotide change	Amino acid change^a^	Protein domain^b^	Feature of deafness^c^	Defects on temporal bone CT	Location	References
del 2.6 kb, 6.5 kb, 7 kb, 4.4 kb	NA	U	NA	NA	Korea	[22]
del 8 kb	NA	U	Mixed	Yes	Korea	[22]
de30 kb	NA	U	Mixed	Yes	Korea	[22]
del 20 kb	NA	U	Mixed	Yes	Korea	[22]
del 130 kb	NA	U	Mixed	Yes	Korea	[22]
del 200 kb	NA	U	Mixed	Yes	Korea	[22]
del 220 kb	NA	U	Mixed	Yes	Korea	[22]
del530 kb	NA	U	SNHL	Yes	US	[23]
del 1200 kb	NA	U	SNHL	Yes	Spain	[24]
del entire gene	NA	Entire gene	Mixed	Yes	Korea	[22]
c.79C *>* T	p.Gln27*	U	SNHL	Yes	Poland,	[19]
c.293C > A	p.Ser98*	U	Mixed	Yes	France	[26]
c.341G > A	p.Trp114*	U	SNHL	Yes	Pakistan	[27]
c.346delG	p.Ala116Profs	U	SNHL, Mixed	Yes	Poland,Turkey	[19, 28]
c.383delG	p.Gly128 fs	U	SNHL	Yes	Korea	[29]
c.406C > T	p.Gln136*	U	SNHL	Yes	Pakistan	[27]
c.499 C > T	p.Arg167*	U	Mixed	Yes	Korea	[30]
c.530C > A	p.Ser177*	U	SNHL	Yes	China	[31]
c.559G *>* T	p.Glu187*	U	SNHL	Yes	Poland,	[19]
601–606delTTCAAA	p.Phe201/Lys202 del	S	Mixed	Yes	Japan	[32]
603-610delCAAA	p.Lys202 fs	S	SNHL	Yes	Netherlands	[5]
c.623 T > A	p. Leu208*	S	SNHL	Yes	Poland,Korea	[19, 29, 33]
c.632C > T	p.Thr211Met	S	Mixed	Yes	Korea	[33]
c.647G > A	p.Gly216 Glu	S	SNHL	Yes	China	[11]
c.648-651delG	p.Arg215 fs	S	Mixed	Yes	Netherlands	[5]
c.650 T *>* A	p.Leu217*	S	SNHL	Yes	Poland	[19]
c.669 T > A	p.Tyr223*	S	SNHL	Yes	China	Present study
c. 683C > T	p.Ser228Leu	S	SNHL	Yes	US	[23]
c.686A > G	p.Gln229Arg	S	SNHL	Yes	Korea	[33]
c.689C > T	p.Thr230Ile	S	Mixed	Yes	US	[34]
c.707A > C	p.Glu236Ala	S	SNHL	Yes	Turkey	[28]
NA	p.Glu236Asp	S	NA	No	France	[26]
c.727_728insA	p.Asn244Lysfs*26	S	SNHL	Yes	Japan	[18]
NA	p.Arg282Gln	H	NA	No	France	[26]
NA	p.Ile285Asn	H	NA	NA	France	[26]
c.772delG	p. Glu 258Argfs	H	SNHL	Yes	Turkey	[28]
c.823C *>* T	p.Gln275*	H	SNHL	Yes	Poland	[19]
c.862del4	p.Ser288Gln fs*37	H	Mixed	Yes	UK	[35]
NA	p.Ser288Cys fs*40	H	NA	No	France	[26]
c.895delA	p.Leu298 fs	H	Mixed	Yes	Netherlands	[5]
c.902C > T	p.Pro301Leu	H	SNHL	NA	Ecuador	[28]
c.907C > T	p.Pro303Ser	H	Mixed	Yes	UK	[25]
c.916C *>* T	p.Gln306*	H	SNHL	Yes	Poland,	[19]
c.923 T *>* A	p.Ile308Asn	H	Mixed	Yes	France	[26]
NA	p.Ile308 Ile fs*28	H	NA	No	France	[26]
c.925 T > C	p.Ser309Pro	H	SNHL	Yes	China	[36]
c.927delCTC	p.Ser310del	H	Mixed	Yes	Korea, China	[37] Present study
c.935C > T	p.Ala312Val	H	SNHL	Yes	UK	[35]
c.950 T > G	p.Leu317Trp	H	Mixed	Yes	Netherlands	[5]
c.950dupT	p. Leu317Phefs*12	H	SNHL	Yes	Korea	[33]
c.967C > G	p.Arg323Gly	H	Mixed	Yes	Korea	[38]
c.971 T *>* A	p.Val324Asp	H	SNHL	Yes	Poland,	[19]
c.973delT	p.Trp325Glyfs*12	H	Mixed	Yes	China	Present study
c.973 T > A	p.Trp325Arg	H	SNHL	Yes	Germany	[39]
c. 983A > C	p.Asn328Thr	H	Mixed	Yes	UK	[25]
c.985C > G	p.Arg329Gly	H	Mixed	Yes	US	[34]
c.986G > C	p.Arg329Pro	H	Mixed	Yes	Korea	[37]
c.987 T > C	p.Leu308Thr	H	SNHL	NA	Nigeria	[28]
c.990A > T	p.Arg330Ser	H	SNHL	Yes	Netherlands	[5]
c.1000A > G	p.Lys334Glu	H	Mixed	Yes	Netherlands	[5]
c.1069delA	p. Thr 354Glnfs*115	D	SNHL	Yes	Korea	[33]
c.1084 T > C	p.X362Argexf*113	D	SNHL	Yes	Korea	[33]

^a^*fs* frameshift, *NA* not available, ^**b**^H and S indicate the POU-homeodomain and POU-specific domain respectively; *U* Upstream, *D* Downstream, ^**c**^*SNHL* sensorineural HL, *Mixed* mixed HL

## Methods

### Clinical evaluation

Patients were enrolled from families 1486, 2741, and ZSJ (three ethnic Han Chinese families) through the Department of Otolaryngology of the General Hospital of the People’s Liberation Army, Beijing, China which collected data and DNA samples from more than 10,000 patients. We chose three families with specific manifestation of temporal bone CT from this cohort. Clinical evaluations, temporal bone imaging results, audiograms, and other relevant clinical information were collected for each family member. The probands had no obvious syndromic symptoms, and *GJB2*, *SLC26A4*, and m.1555A > G and m.1494C > T mutations in mtDNA *12S rRNA* were excluded. Genomic DNA was extracted from peripheral blood using a blood DNA extraction kit according to the protocol provided by the manufacturer (Tiangen Biotech, Beijing, China).

### Families 1486, 2741, and ZSJ

**Fig. 1:**
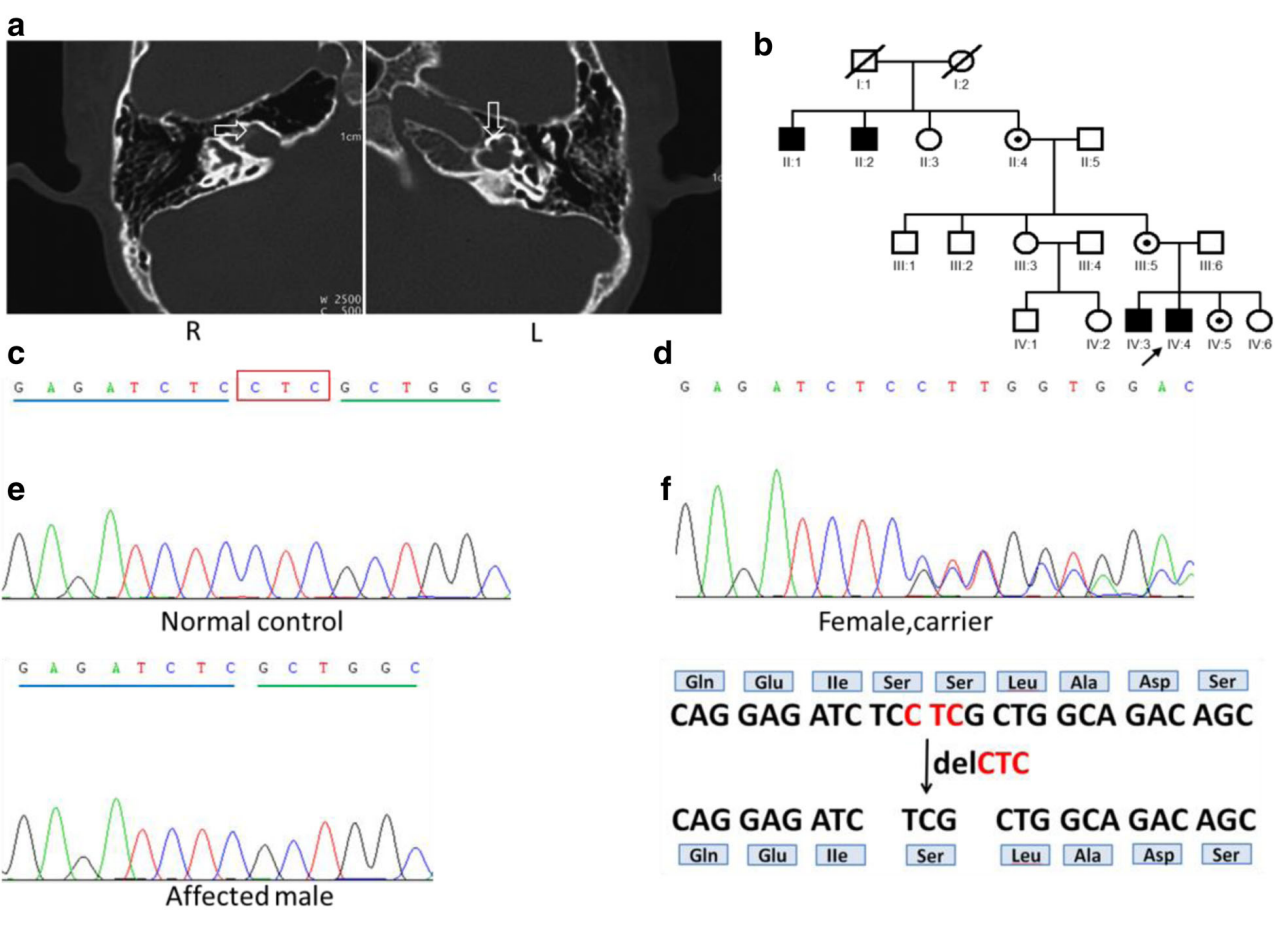
Pedigree, clinical phenotypes, and mutation analysis in family 1486. **a** Temporal bone computed tomography (CT) images of the proband of family 1486 demonstrating dilation of the lateral end of the internal acoustic meatus (IAM) and a malformed cochlea; the basal turn of the cochlea was incompletely separated from the IAM (arrow); **b** Pedigree of Family 1486 with multiple congenital profound sensorineural hearing impairment cases (Affected subjects are denoted in black. Arrow indicates the proband. Mutation carrier are denoted with dot within a symbol); **c** Wild-type sequence of *POU3F4* including sites 927–929; **d** A heterozygous c.927delCTC mutation was found in the female carriers; **e** A hemizygotic c.927delCTC mutation was detected in the affected males; **f** Amino acid changes caused by changes in the DNA sequence. A three-nucleotide deletion (from position 927 to 929) in the coding region of *POU3F4* results in the deletion of serine at position 310

Family 1486 is a four-generation Chinese family, and the pedigree of this family is consistent with an X-linked inheritance pattern (Fig. 1b). Fifteen family members, including four patients (4 males: II:1, II:2, IV:3, and IV:4) with HL and 11 individuals (5 males: II:5, III:1, III:4, III:6, and IV:1, and 6 females: II:4, III:3, III:5, IV:2, IV:5, and IV:6) with normal hearing participated. The medical histories of the participants were obtained through structured questionnaires, otological examinations, and systematic assessments for signs of syndromic deafness. The proband underwent temporal bone CT scans and auditory brainstem responses. Pure tone audiometry was not available for the proband because of his young age.

**Fig. 2:**
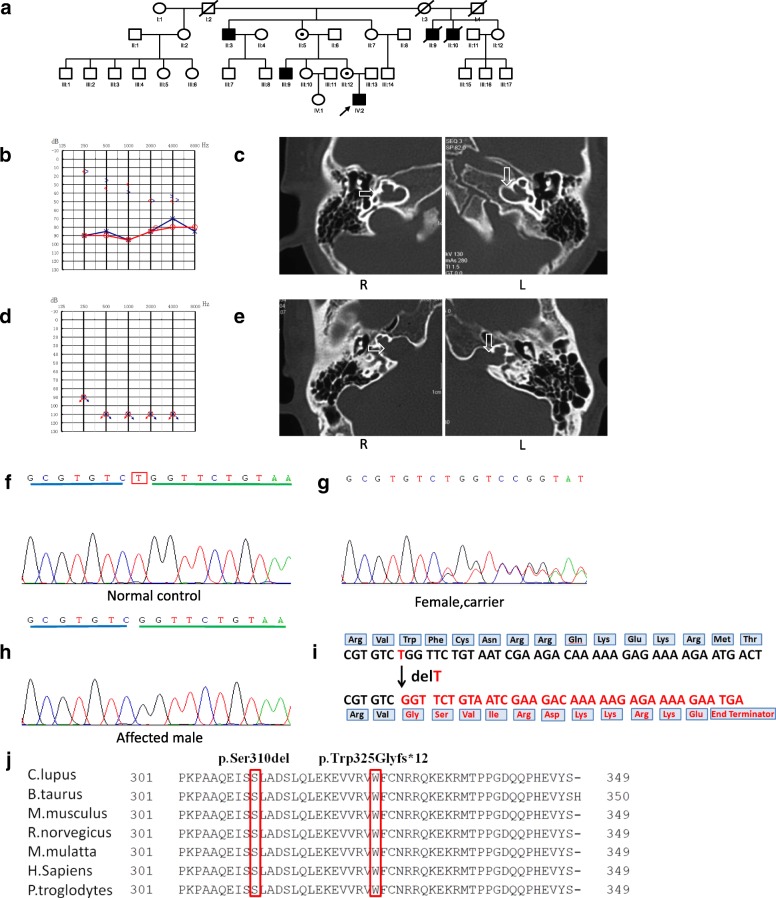
Pedigree, clinical phenotypes and mutation analysis in family 2741. **a** Pedigree of family 2741 with congenital mixed hearing impairment and sensorineural hearing impairment cases (Affected subjects are denoted in black. Arrow indicates the proband. Mutation carrier are denoted with dot within a symbol); **b** Audiograms of both ears for the proband, who exhibited typical audiometric features of mixed hearing impairment; **c** Temporal bone CT images of the proband demonstrating dilation of the bottom of the IAM and a deficit in the bony plate, which separates the basal turn of the cochlea and the IAM (arrow); **d** Audiograms of both ears from the uncle of the proband, who shows profound sensorineural hearing impairment; **e** Temporal bone CT images of the uncle of the proband demonstrating dilation of the lateral end of the IAM and bone deficiency between the basal turn of the cochlea and the IAM (arrow). **f** Wild-type sequence of *POU3F4* including position 973; **g** A heterozygous c.973delT mutation was found in the female carriers; **h** A hemizygotic c.973delT mutation was detected in the affected males; **i** Amino acid change caused by changes in the DNA sequence leading to a predicted frameshift mutation and truncation of the POU3F4 protein; **j** Panel 1 marks the position of the c.973delT (p.Trp325Glyfs*12) mutation and panel 2 marks the position of the c.927delCTC (p.Ser310del) mutation. The POU homeodomain (from Gly276 to Arg335) is highly conserved in different species

Family 2741 is also a four-generation Chinese family, and the pedigree of this family showed a typical X-linked recessive inheritance pattern of HL (Fig. 2a). Ten family members were assessed: three patients (three males: II:3, III:9, and IV:2) with HL and seven individuals (two males: II:6 and III:13, and five females: II:5, II:7, II:12, III:10, and III:12) with normal hearing. The medical histories of the participants were obtained through structured questionnaires, otological examinations, and systematic assessments for signs of syndromic deafness. We obtained 10 blood samples (II:3, II:5, II:7, II:6, II:12, III:9, III:10, III:12, III:14, and IV:2) and the affected boy (IV:2) and his uncle (III:9) underwent temporal bone CT scans. Due to long distances, no temporal bone CT image was obtained from the affected boy’s granduncle (II:3). The proband presented with mixed HL, his uncle had profound sensorineural HL, and the females presented with normal hearing.

**Fig. 3:**
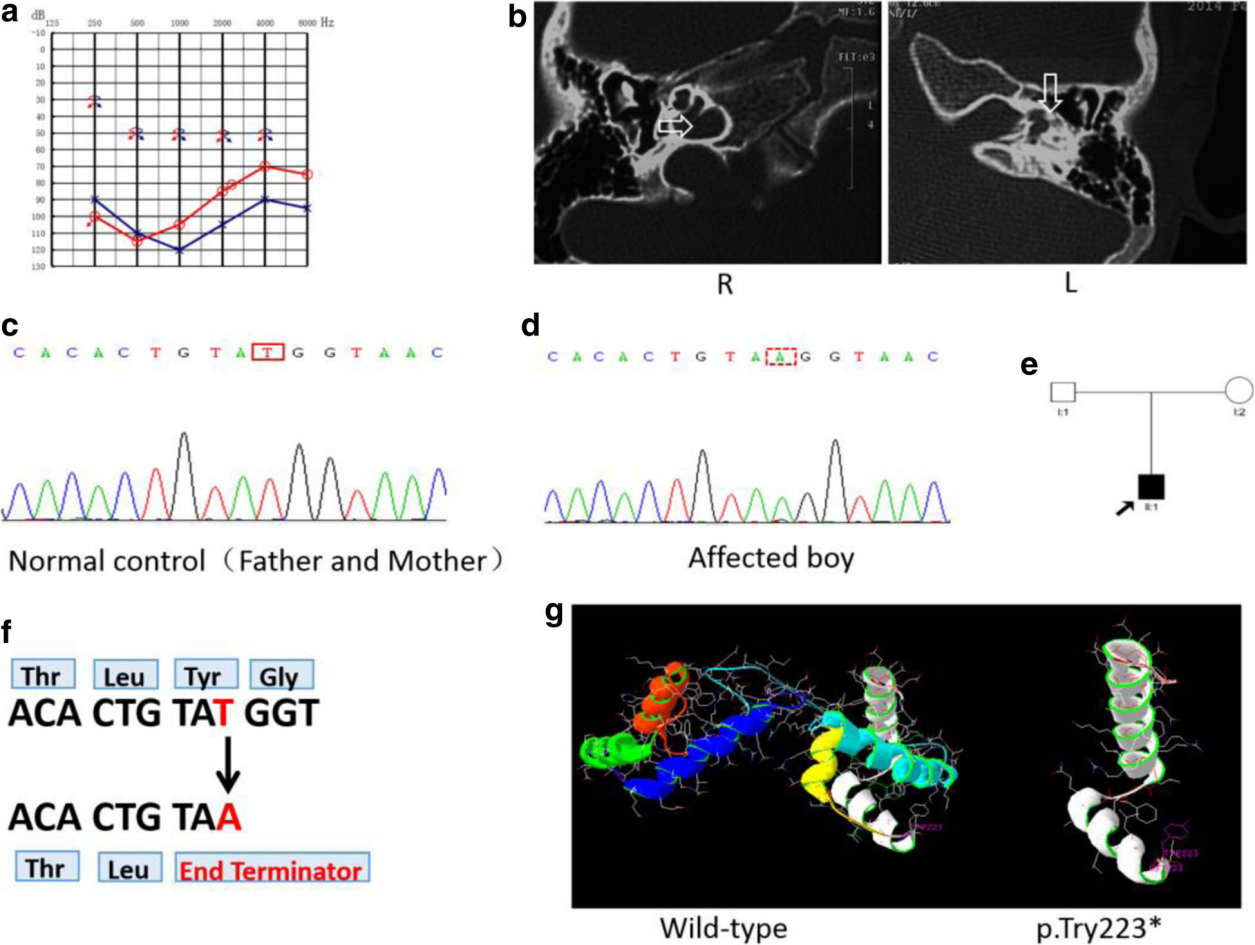
Pedigree, clinical phenotypes and mutation analysis in family ZSJ. **a** Audiograms of both ears from the proband exhibited profound sensorineural hearing impairment; **b** Temporal bone CT images of the proband demonstrating dilation of the lateral end of the IAM and a deficit in the basal turn of the cochlea in the right ear (arrow) in addition to dilation of the lateral end of the IAM and an incompletely developed cochlea in the left ear (arrow); **c** Wild-type sequence of *POU3F4*, including site 669; **d** A hemizygotic c.669 T > A mutation was detected in the affected boy; **e** Pedigree of family ZSJ; **f** Stop codon caused by changes in the DNA sequence; **g** Molecular modeling of wild-type and mutant POU3F4 proteins. The c.669 T > A mutant creates a new stop codon and is predicted to result in a truncated protein lacking normal POU3F4 transcription factor function

In family ZSJ, the proband was a 5-year-old boy with congenital inner ear malformation and profound HL, but no one else in his family had HL, the inheritance pattern is unclear (Fig. 3e). Because of minimal progression in auditory ability after wearing hearing aid for 3 years and profound HL, the boy underwent cochlear implant surgery on his left ear at Chinese PLA General Hospital. A physical and otoscopic examination, temporal bone CT scans, and audiological studies were performed before surgery. According to the radiological and hearing findings, we suspected that he had DFNX2, and a mutation analysis of the *POU3F4* gene was performed.

### Sequencing analysis of POU3F4

Genomic DNA was extracted from blood using a DNA Extraction Kit (Tiangen Biotech). Briefly, the entire coding region and splice sites of the single exon of *POU3F4* (NM_000307.1) were amplified in three overlapping fragments using the following three pairs of forward (F) and reverse (R) primers: 5’-ACTTCCTGCTTGGGTCTCATTG (F1) and 5’-GGAGTGATCCTGGCAATGGT (R1), 5’-GGCACCGAACCCGTCTATC (F2) and 5’-TCCCCTGGCGGAGTCAT (R2), and 5’-TTGGAGAAGGAAGTGGTGCG (F3) and 5’-CCCAGCTTGGACTGCTTAATGTA (R3). Polymerase chain reaction (PCR) amplification was performed in a total volume of 20 μL: 2 μL of 10× buffer, 0.5 μL of primer L, 0.5 μL of primer R, 0.5 μL of deoxynucleotide triphosphates, 0.2 μL of Taq polymerase, 1 μL of DNA, and 15.3 μL of water. PCR began with incubation at 95 °C for 5 min, followed by nine cycles of denaturation for 45 s at 95 °C, annealing for 45 s at 58 °C, and extension for 30 s at 72 °C; this was followed by 34 cycles of denaturation for 45 s at 95 °C, annealing for 45 s at 55 °C, extension for 30 s at 72 °C, and a final 7-min extension at 72 °C. PCR products were resolved by gel electrophoresis to confirm product amplification. Sequencing was performed using the ABI 3100 Avant Capillary Electrophoresis System (Applied Biosystems, Foster City, CA, USA). We also sequenced 100 Chinese individuals with normal hearing to determine whether the mutations were present in the unaffected Chinese population.

## Results

### Clinical features of the family

In family 1486, the proband and the affected male members exhibited congenital severe-to-profound sensorineural hearing impairment. Auditory brainstem response thresholds with clicks were 70 dB in both ears of the proband. In family 2741, the proband exhibited typical audiometric features of mixed hearing impairment (Fig. 2b), while the maternal uncle of the proband was completely deaf (Fig. 2d). In family ZSJ, the proband exhibited profound sensorineural deafness (Fig. 3a).

Temporal bone computed tomography (CT) was performed in four patients from three families; identical findings were observed: an absent modiolus, and the basal turn of the cochlea was incompletely separated from the IAM and appeared to be a continuation of the IAM. Typically, a bulbous dilatation of the lateral end of the IAM was identified (Figs. 1a, 2c, e, and 3b: R) (arrow). All of the findings were symmetrical, except that the left cochlea of the affected boy in family ZSJ was not fully developed and smaller than the cochlea of the other side (Fig. 3e).

Cochlear implantation with the Nucleus® Slim Straight Electrode was performed in the proband from family ZSJ. Due to widening of the bony IAM during cochlear implantation, cerebrospinal fluid (CSF) “gusher” was observed upon opening the round window, and a piece of prepared muscle tissue was used to block the leakage after inserting the electrodes. CSF leakage did not occur after surgery in this case. Intra-operative CT was utilized to ensure correct electrode positioning and to prevent the electrode from entering the IAM. The postoperative pure tone audiograms under aided conditions showed hearing thresholds of 60 dB.

### Identification of POU3F4 mutations in families 1486, 2741, and ZSJ with DFNX2

In family 1486, the c.927delCTC (p.Ser310del) mutation was identified in the males (IV:3 and IV:4) with profound HL, consistent with X-linked inheritance. A three-nucleotide deletion (from position 927 to 929) in the coding region of *POU3F4* resulted in the deletion of a serine at position 310 within the POU homeodomain (Fig. 1e), without affecting the coding frame (Fig. 1f). The mother (III:5), grandmother (II:4), and sister (IV:5) were heterozygous for c.927delCTC (Fig. 1d).

In family 2741, the 10 family members were enrolled in the study, 3 of whom were classified as affected (consistent with X-linked inheritance) (Fig. 2a). The c.973delT (p.Trp325Glyfs*12) mutation was identified in these three male patients, leading to a predicted frameshift mutation and truncation of the protein. This mutant protein lacks part of the *POU3F4* protein, including the POU homeodomain, which is highly conserved across species (Fig. 2j). Sequence analysis of all family members revealed a deletion at nucleotide position 973 in the patient (IV:2, II:3 and III:9; Fig. 2h), and the mother (III:12) and grandmother (II:5) were heterozygous for c.973delT (Fig. 2g). Mutation screening of *POU3F4* in families 2741 and 1486 showed that p.Trp325Glyfs*12 and p.Ser310del, respectively, co-segregated in all affected males examined. Heterozygous p.Trp325Glyfs*12 and p.Ser310del were also found in the female carriers, separately. Moreover, neither mutation was observed in any of the 100 unrelated controls with normal hearing by direct sequencing. According to the standards and guidance in the 2015 American College of Medical Genetics and Genomics (ACMG), two variants identified in this study, c.973delT and c.927delCTC (p.Trp325Glyfs*12 and p.Ser310del), are pathogenic variants, not rare polymorphisms, and are among the most conserved amino acids in the POU homeodomain (Fig. 2j).

In family ZSJ, sequence analysis of *POU3F4* in the affected boy revealed a de novo transversion, c.669 T > A (Fig. 3d), resulting in a nonsense mutation (p.Tyr223*, Fig. 3f) and the creation of a new stop codon. Thus, it is predicted to result in a truncated protein lacking normal POU3F4 transcription factor function. Neither parent carried the c.669 T > A mutation (Fig. 3c). To determine the reliability of the de novo mutation, a paternity test was used to confirm the biological relationship between the parents and the boy. Moreover, the mutation was not found in any of the 100 unrelated controls with normal hearing by direct sequencing. Molecular modeling showed that the tyrosine residue at position 223 is located at the end of the second helix of the specific homeodomain. The c.669 T > A mutant is predicted to result in the deletion of the third helix of the specific homeodomain and all three helices of the POU homeodomain, which creates a truncated protein lacking normal POU3F4 transcription factor function (Fig. 3g).

Two single nucleotide polymorphisms, Ala708Gly and Gly710Cys (rs5921978 and rs5921979, respectively), were also observed in all subjects of the families and controls, indicating polymorphisms (data not shown).

**Fig. 4:**
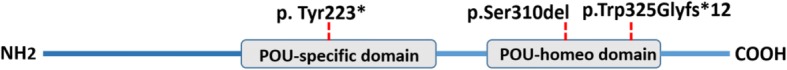
Schematic illustration of the POU3F4 protein. In this study, three mutations were identified: the p.Tyr223*mutation located in the POU-specific domain, and the p.Trp325Glyfs*12 and p.Ser310del mutations located in the POU homeodomain

In this study, three mutations were identified: the p.Tyr223* mutation located in the POU specific-domain, and the p.Trp325Glyfs*12 and p.Ser310del mutations located in the POU homeodomain (Fig. 4).

## Discussion

Deafness segregating at the DFNX2 locus is associated with mutations in the *POU3F4* gene. The human POU3F4 protein contains a POU-specific domain, with a length of 67 amino acids (from Lys194 to Asp260), a linker of 15 residues (from Ser261 to Gln275), and a POU homeodomain, with a length of 60 amino acids (from Gly276 to Arg335) [17]. Previous studies have suggested that HL in DFNX2 is caused by loss-of-function of the POU3F4 protein rather than gain-of-ectopic functions in the mutant proteins.

Previous studies have identified more than 60 mutations invariably located in the POU- specific and homeodomains of *POU3F4*. In this study, we identified three mutations in the *POU3F4* gene in two Chinese families displaying X-linked inheritance HL and one sporadic case with indefinite inheritance pattern. Two of the mutations (p.Trp325Glyfs*12 and p.Ser310del) occurred in the POU homeodomain and the third mutation (p.Tyr223*) was identified as a de novo mutation occurring in the specific homeodomain, which caused a premature termination resulting in a protein lacking part of the specific homeodomain and the entire POU homeodomain. Usually, diagnosing the disorder in sporadic cases with inner ear malformation is difficult. In this report, *POU3F4* gene sequencing identified a “novel *de novo*” nonsense mutation (c.669 T > A). This is the third reported case in which such a disorder occurred in the affected individual due to a spontaneous de novo mutation not inherited from the parents. Two previously reported *POU3F4* de novo mutations were p.Asn244Lysfs*26 [18] and p.Leu217* [19]. We believe that many more such patients with this disorder are likely to be diagnosed in the near future due to a combination of clinical features and genetic testing.

Individuals, usually males, with variants in this gene exhibit characteristic clinical and radiological features. The most frequent form of X-linked deafness, DFNX2, is characterized by temporal bone abnormalities, stapes fixation and, in most cases, a mixed type of deafness. All of the probands from the three families examined herein showed characteristic inner ear radiological features compatible with incomplete partition type III. The HL in these individuals can be mixed, with the sensorineural component usually presenting in infancy and showing progression with age. Temporal bone CT scans showed dilatation of the lateral end of the IAM and/or a bone deficiency between the basal turn of the cochlea and the IAM. The conductive HL component, which may or may not be present, is due to fixation of the stapes. Because of outward pressure of perilymphatic fluid on the oval window coupled with defects in the size and shape of the stapes footplate further compromise ossicular movement and, collectively, these anomalies result in progressive sensorineural deafness in patients with DFNX2. In patients with radiological abnormalities of the cochlea on CT scans like this, a perilymphatic flow (or “gusher”) can occur during inner ear surgery [20], which will result in immediate deafness along with concomitant complaints of vertigo and tinnitus. Preoperative evaluation before stapes or cochlear implant surgery is very important. In our cases, an expected CSF gusher was seen in the patient (family ZSJ) when the round window was opened. Cochleostomy was sealed with muscle tissue, and there was no CSF leakage, meningitis, or facial stimulation after surgery. Saeed et al. encouraged additional surgical obliteration of the middle ear space and external auditory canal to avoid persistent CSF leakage and its associated complications [21]. However, in our cases, this was not necessary.

The management of patients with DFNX2 depends on the degree of the overall HL. If the HL is a milder conductive or sensorineural hearing impairment, hearing aids are often a first-line recommendation. Some patients with bilateral mixed HL but serviceable bone conduction thresholds benefit from bone-anchored hearing aid technologies. However, patients with severe-to-profound hearing impairment can benefit from cochlear implant surgery. Kang et al. compared the audiologic performance of patients with X-linked deafness after cochlear implantation to those with a normal inner ear structure after implantation and found no significant difference between the two groups. The patient who underwent cochlear implantation described in this article had postoperative hearing thresholds of approximately 60 dB at 12 and 24 months after activation of the cochlear implant. We believe that the limited auditory perception and language acquisition were due to serious malformation of the cochlea. Regardless, with thoughtful preparation and the assistance of intraoperative imaging, cochlear implantation in patients with DFNX2 can be performed safely.

Several studies have reported HL in female siblings or mothers of affected males with mutations in *POU3F4*. In 2009, Marlin et al. reported the phenotype of eight independent females from families which male carriers presenting with typical DFNX2 and carrying *POU3F4* variants, and in which three female carriers have hearing loss [35]. However, we did not observe HL in the heterozygous mothers in our two families (families 1486 and 2741).

## Conclusions

The identification of pathogenic alleles causing X-linked recessive deafness will improve molecular diagnosis, genetic counseling, and knowledge of the molecular epidemiology of HL among Chinese individuals. Taking these results together, we recommend preoperative gene mutation analysis in patients who have DFNX2 diagnosed on the basis of characteristic radiological findings. If a genetic cause of HL is determined, families with hereditary HL can be provided with prognostic information, the risk of recurrence, and improved rehabilitation options.

## Abbreviations

ACMG: American College of Medical Genetics and Genomics
CT: Computed tomography
DFNX2: X-linked inheritance deafness-2
HL: Hearing loss
IAM: Internal acoustic meatus
*POU3F4*: POU domain class 3 transcription factor 4
